# The demographic impact and development benefits of meeting demand for family planning with modern contraceptive methods

**DOI:** 10.1080/16549716.2018.1423861

**Published:** 2018-02-08

**Authors:** Daniel Goodkind, Lisa Lollock, Yoonjoung Choi, Thomas McDevitt, Loraine West

**Affiliations:** a U.S. Census Bureau, Population Division, Washington, DC, USA; b Johns Hopkins Bloomberg School of Public Health, Baltimore, MD, USA; c Independent Researcher, Burke, VA, USA

**Keywords:** Demographic dividend, United Nations SDGs (sustainable development goals), reproductive health, biometric models, population projections, sub-Saharan Africa

## Abstract

**Background**: Meeting demand for family planning can facilitate progress towards all major themes of the United Nations Sustainable Development Goals (SDGs): people, planet, prosperity, peace, and partnership. Many policymakers have embraced a benchmark goal that at least 75% of the demand for family planning in all countries be satisfied with modern contraceptive methods by the year 2030.

**Objective**: This study examines the demographic impact (and development implications) of achieving the 75% benchmark in 13 developing countries that are expected to be the furthest from achieving that benchmark.

**Methods**: Estimation of the demographic impact of achieving the 75% benchmark requires three steps in each country: 1) translate contraceptive prevalence assumptions (with and without intervention) into future fertility levels based on biometric models, 2) incorporate each pair of fertility assumptions into separate population projections, and 3) compare the demographic differences between the two population projections. Data are drawn from the United Nations, the US Census Bureau, and Demographic and Health Surveys.

**Results**: The demographic impact of meeting the 75% benchmark is examined via projected differences in fertility rates (average expected births per woman’s reproductive lifetime), total population, growth rates, age structure, and youth dependency. On average, meeting the benchmark would imply a 16 percentage point increase in modern contraceptive prevalence by 2030 and a 20% decline in youth dependency, which portends a potential demographic dividend to spur economic growth.

**Conclusions**: Improvements in meeting the demand for family planning with modern contraceptive methods can bring substantial benefits to developing countries. To our knowledge, this is the first study to show formally how such improvements can alter population size and age structure. Declines in youth dependency portend a demographic dividend, an added bonus to the already well-known benefits of meeting existing demands for family planning.

## Background

In 2015, member states of the United Nations endorsed a set of Sustainable Development Goals (SDGs), organized around five themes: people, planet, prosperity, peace, and partnership [1]. Among the many proposals endorsed, one stands out for offering progress on all five of these themes: the improvement in access to family planning, a component under the broader target of sexual and reproductive health (see SDG target 3.7) [2]. At its broadest, improved access to family planning options helps advance human rights for all regardless of age, sex, marital status, and health. Informed and voluntary decision making related to family planning contribute towards women’s education, empowerment, gender equality, and human capital development [2]. Such goals provide important nodes for international cooperation. And in the arena of public health, family planning provides numerous benefits, such as the reduction of maternal and child mortality [3–5] as well as the prevention of HIV transmission [6].

**Figure 1.**
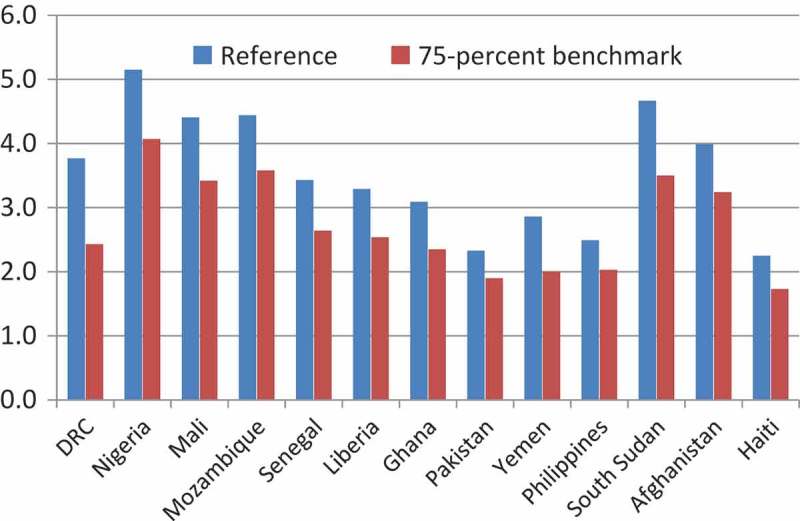
Total fertility rate in 2030: Reference and 75% benchmark projections. The total fertility rate (TFR) is the expected number of births per woman’s reproductive lifetime.

**Figure 2.**
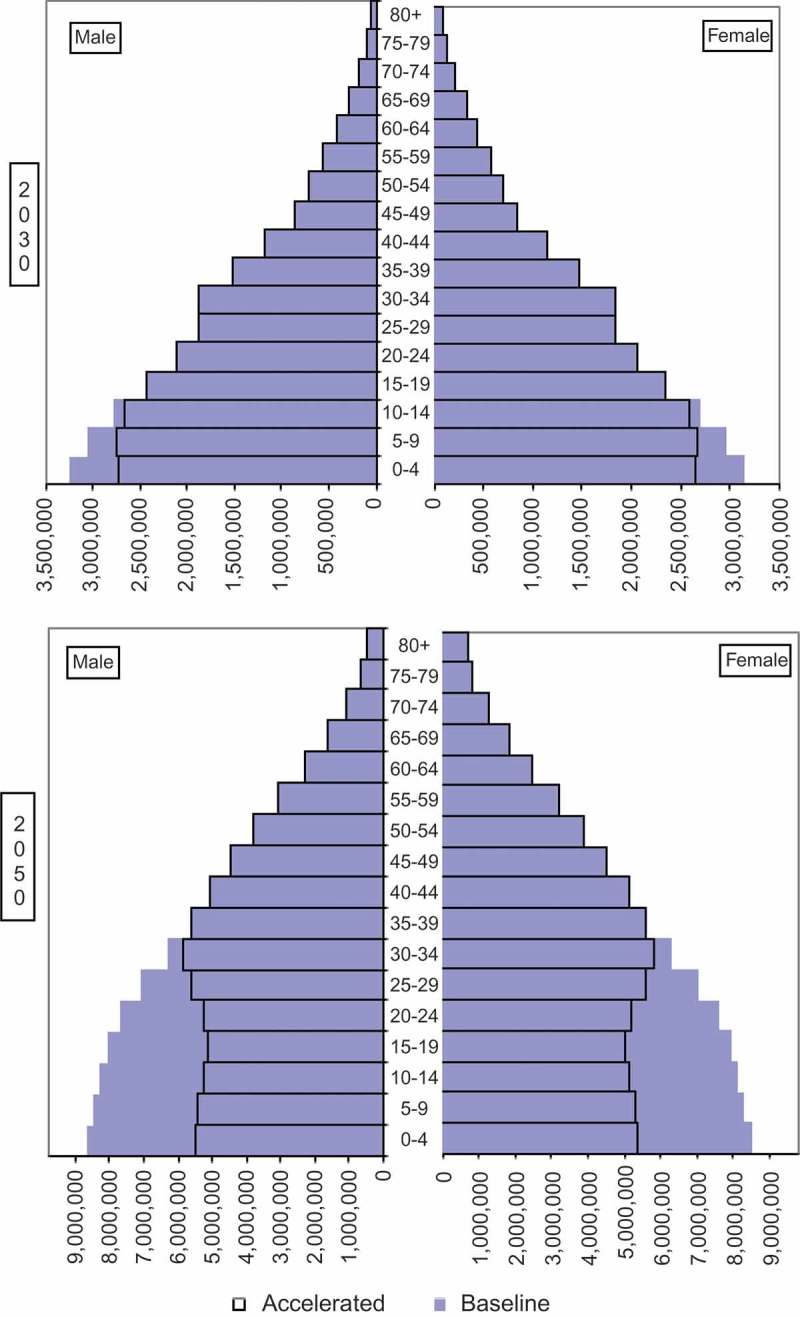
Democratic Republic of Congo, populaton by age and sex in 2030 and 2050: reference and 75% benchmark projections.

Family planning facilitates many of these goals by reducing unwanted fertility and altering population dynamics. The pace of population growth has a major influence on the environment, food security, poverty elimination, and sustainable development [2]. Changing age structures from rapid fertility reduction can also accelerate economic growth [2,7]. In light of all this, efforts to realize the family planning component of the SDGs can have any number of positive benefits. To monitor progress in family planning, a new indicator – percent of demand for family planning satisfied with modern contraception, hereinafter referred to as ‘met demand’ – as well as an associated benchmark (that this indicator reach at least 75% by 2030) have been adopted under the SDGs [8–10]. This indicator is appealing compared to other indicators (such as proportion using contraception) because, instead of trying to create demand for contraception, success is measured in satisfying the demand that is already projected to exist. Countries vary widely in both their current and future expected levels of met demand [11]. In order to achieve the 2030 benchmark, on average, low- and middle-income countries need to double currently projected rates of progress [12].

This study estimates the demographic impact of achieving the benchmark for 13 countries that seem furthest from meeting that benchmark. Specifically, we present a set of demographic indicators for each country – total fertility rate (TFR), population size, growth rate, proportions of population by age group, and child dependency ratios – in 2030 and 2050 based in part on two projections of modern contraceptive prevalence rates (MCPRs): the current projections by the United Nations and projections which assume that each country instead met the 75% benchmark (hereinafter referred to as the reference and benchmark projections, respectively). Falling child dependency ratios provide an especially valuable window into the potential economic benefits (demographic dividend) [13] of improved fulfillment of existing demands for contraception.

## Methods

### Study countries

The 13 countries expected to be furthest from reaching the benchmark were determined based on United Nations’ projections [11]. They were chosen from among the 24 high-priority countries identified by the US Agency for International Development (USAID) for family planning assistance and span across three continents. The 13 countries include Afghanistan, Democratic Republic of Congo (DRC), Ghana, Haiti, Liberia, Mali, Mozambique, Nigeria, Pakistan, the Philippines, Senegal, South Sudan, and Yemen. Table 1 shows the current and future projected prevalence of modern contraceptive use under the baseline (columns 1 and 3), the projected prevalence if the 75% benchmark was met (column 5), and the increase in the MCPR in each country. On average, meeting the benchmark would imply a 16 percentage point increase in the MCPR by 2030.

**Table 1.** Reference and 75% benchmark modern contraceptive prevalence rate (MCPR) scenarios for 13 countries: 2014 and 2030.

Country	1) Estimated MCPR 2014	*2) Percent of demand met with modern methods 2014*	3) Projected MCPR 2030, reference model (United Nations)	*4) Percent of demand met with modern methods 2030, reference model (United Nations)*	5) Projected MCPR 2030, if 75% of demand benchmark was achieved	6) Projected increase in MCPR 2030 over reference, if 75% of demand benchmark was achieved
South Sudan	2.2	*6.1*	15.5	*29.0*	40.1	24.6
DRC	7.7	*15.2*	18.0	*30.0*	45.1	27.1
Nigeria	10.1	*27.2*	20.8	*41.9*	37.2	16.4
Mali	10.9	*26.7*	21.6	*41.9*	38.6	17.0
Mozambique	14.9	*33.8*	33.1	*54.7*	45.4	12.3
Senegal	16.1	*33.7*	26.9	*46.4*	43.5	16.6
Liberia	19.0	*35.7*	29.7	*48.8*	45.7	16.0
Ghana	19.7	*34.5*	30.4	*47.8*	47.7	17.3
Afghanistan	23.1	*41.0*	40.4	*58.4*	51.9	11.5
Pakistan	26.8	*46.0*	41.5	*59.4*	52.4	10.9
Yemen	27.8	*39.8*	41.8	*54.2*	57.8	16.0
Haiti	32.8	*46.3*	44.1	*58.6*	56.5	12.4
Philippines	38.0	*52.1*	45.5	*59.9*	57.0	11.5

Note: the ordering of countries is by estimated MCPR in 2014. MCPRs refer to married women.

Sources: 11, 15.

#### Data

For each country, we began with the United Nation’s median estimates of MCPR, traditional contraceptive prevalence rate (TCPR), and total demand for family planning among married women through 2030 [11]. United Nation’s projections of such measures are based on a Bayesian hierarchical model and a modeling dataset comprised of estimates from population-based surveys since 1950 from around the world. Unmet need for family planning is modeled based on a statistical relationship observed in survey estimates of MCPR and unmet need [14]. Total demand refers to the percent of women who wants to delay, space, or limit pregnancy in the next 2 years. The sum of MCPR, TCPR, and unmet need identifies potential demand for family planning [8,10].

Method-specific contraceptive prevalence rates were drawn from either the United Nations wallchart on contraceptive patterns [15] or the latest Demographic and Health Survey in each country, whichever was more recent. The same relative mix among modern methods was assumed in 2014, the baseline of our projections. All other demographic variables for the baseline (population size, age-sex structure, as well as age-specific fertility, mortality, and net migration) came from the International Data Base (IDB) [16].

#### Translating contraceptive use into fertility rates and population projections

For each country, three steps are required to assess the demographic impact of achieving the benchmark of meeting 75% of the demand for family planning with modern contraception by 2030. First, future expected contraceptive prevalence in both reference and benchmark projections are translated into levels of fertility. Second, these levels of fertility are incorporated into a pair of population projections. Third, the resulting reference and benchmark projections are compared.

### Step 1: translating contraceptive use into estimated and projected fertility rates

Contraception is one factor among several that influences fertility. The key ‘proximate determinants’ of fertility also include marital patterns, post-partum sterility, and other factors [17,18]. A full biometric model for translating such factors into the number of expected births per woman (or TFR) is shown below. Total fecundity (TF) indicates the theoretical maximum expected TFR (roughly 15 births per woman). That maximum is reduced based on indexes for aforementioned factors that range from one to zero. Each index is constructed such that it falls progressively below one as changes in that factor have a greater inhibiting effect on fertility (e.g. contraceptive use increases and age at marriage rises). TF is multiplied by each of the indexes, and the estimated TFR will be lower to the extent that the product of indexes is below one.

(1) $$TFR=\left[C_{m}*C_{c}*C_{i}*C_{a}*C_{p}*TF\right]$$

where:

C_m_ is an index of marriage (proportion of women ages 15–49 in union)

C_c_ is the index of contraception

C_i_ is the index of postpartum insusceptibility

C_a_ is the index of abortion

C_p_ is an index of infecundity or pathological sterility

TFR is the total fertility rate (average expected births per woman)

TF is total fecundity (maximum potential TFR – about 15 births per woman)

The above biometric identity holds at different points in time. Thus, the ratio of the TFR to the proximate determinant indexes in 2014 and 2030, for example (below), can be expressed as:

(2) $$TFR_{2030}/{\left[C_{m}*C_{c}*C_{i}*C_{a}*C_{p}*TF\right]}_{2030}=TFR_{2014}*/{\left[C_{m}*C_{c}*C_{i}*C_{a}*C_{p}*TF\right]}_{2014}$$

where (in our operationalization of the model, to be discussed shortly):

TFR_2014_ is an estimate of the TFR in 2014 drawn from the Census Bureau’s International Data Base (IDB)

TFR_2030_ is the biometric model estimate of the TFR in 2030 from Equation (1)

C indexes (for 2014 and 2030) are included where available

Upon rearranging the equation (below), the TFR in 2030 can be derived from current fertility estimates as well as current and future expected values of the proximate determinant indexes.

(3) $$TFR_{2030}=TFR_{2014}*{\left[C_{m}*C_{c}*C_{i}*C_{a}*C_{p}*TF\right]}_{2030}\;\;/{\left[C_{m}*C_{c}*C_{i}*C_{a}*C_{p}*TF\right]}_{2014}$$

For the countries of interest, not enough information was available to estimate and project indexes for abortion (C_a_) or pathological sterility (C_p_). Thus, for most countries, we used the following abridged biometric model:

(4) $$TFR_{2030}=TFR_{2014}*{\left[C_{m}*C_{c}*C_{i}*TF\right]}_{2030}/{\left[C_{m}*C_{c}*C_{i}*TF\right]}_{2014}$$

The contraceptive index (C_c_) is calculated based on MCPR, TCPR, relative mix of modern methods, and effectiveness of each method to prevent pregnancy [19,20]. For the reference projections, the MCPR and TCPR in 2030 were taken from recent projections by the United Nations. For the benchmark projections, MCPR in 2030 was calculated to be 75% of the United Nation’s projections of potential demand for family planning. TCPR in 2030 also came from the UN projections. In both projections, the relative mix of modern methods and method-specific effectiveness were assumed unchanged between 2014 and 2030.

To provide a better idea of the derivation of some key inputs for the above equations, Table 1 shows the estimated MCPR as well as the percent of the demand for family planning that is satisfied by modern methods in 2014 (columns 1 and 2). The next two columns show the same pair of statistics as of 2030 based on United Nations’ projections of both modern contraceptive use and overall demand for family planning. The fifth column shows the MCPR in 2030 if 75% of the demand for family planning (not shown) was satisfied through modern methods, and the sixth column shows the related percentage point increase in MCPR under the benchmark. The expected increase in MCPR by 2030 is largest (around 25 percentage points or higher in Sudan and DRC) in countries that currently have the lowest MCPR.

The other indexes (C_m_ and C_i_) in 2030 were estimated based on the trajectory of such indexes as indicated by consecutive sources at two points in time, typically from Demographic and Health Surveys (DHSs). Because such data were not available in some countries (Afghanistan, South Sudan, and Yemen), a further abridgement was required to the biometric equation in these countries (see simplified model below). The abridged Equations (4 and 5) to estimate the TFR in 2030 implicitly assume that the other non-modeled indexes do not change over time.

(5) $$TFR_{2030}=TFR_{2014}*{\left[C_{c}\right]}_{2030}/{\left[C_{c}\right]}_{2014}$$

The resulting TFR estimates under the reference and benchmark projections appear in Figure 1 and Table 2. By definition, the higher MCPRs under the latter produce lower fertility rates.

**Table 2.** Reference and 75% benchmark total fertility rate and population growth rate: 2014, 2030, and 2050.

	Baseline scenario						75% benchmark scenario					
	Total fertility rate			Growth rate			Total fertility rate			Growth rate		
*Country*	2014	2030	2050	2014	2030	2050	2014	2030	2050	2014	2030	2050
South Sudan	5.43	4.67	3.49	4.12	2.81	2.14	5.43	3.50	2.23	4.12	2.14	1.30
DRC	4.80	3.77	2.67	2.50	2.23	1.55	4.80	2.43	2.01	2.50	1.36	0.97
Nigeria	5.25	5.15	5.01	2.52	2.81	2.95	5.25	4.07	2.70	2.52	2.18	1.52
Mali	6.16	4.41	2.61	2.66	2.24	1.38	6.16	3.42	2.10	2.66	1.63	0.94
Mozambique	5.27	4.44	3.28	2.44	2.54	2.14	5.27	3.58	2.27	2.44	2.00	1.45
Senegal	4.52	3.43	2.42	2.47	2.00	1.32	4.52	2.64	2.04	2.47	1.48	0.98
Liberia	4.81	3.29	2.22	2.52	1.92	1.20	4.81	2.54	2.02	2.52	1.43	0.97
Ghana	4.09	3.09	2.30	2.19	1.72	1.22	4.09	2.35	2.01	2.19	1.23	0.92
Afghanistan	5.43	3.99	2.58	2.29	2.02	1.33	5.43	3.24	2.13	2.29	1.59	0.97
Pakistan	2.86	2.33	1.93	1.49	1.31	0.69	2.86	1.90	1.71	1.49	1.01	0.46
Yemen	4.09	2.86	1.97	2.72	1.86	1.02	4.09	2.00	1.71	2.72	1.25	0.72
Haiti	2.79	2.25	1.86	1.08	0.96	0.39	2.79	1.73	1.70	1.08	0.58	0.08
Philippines	3.06	2.49	2.13	1.81	1.31	0.79	3.06	2.03	2.00	1.81	1.00	0.55

The ordering of countries is by estimated MCPR in 2014 (Table 1). Total fertility rates for South Sudan, Afghanistan, and Haiti – given data limitations – were estimated based on Equation (5). Total fertility rates for other countries were based on Equation (4).

Although United Nations’ estimates of contraceptive use and demand for family planning are only available through 2030, to model the longer-term impact of achieving the 75% benchmark, we project fertility forward to 2050 using a logistic model based on fertility estimates in 2014, 2020, and 2030 (the TFR in 2020 was derived from the above biometric equations using United Nations’ projections for 2020). The key TFR estimates for each country and each scenario in 2014, 2030, and 2050 are shown on Table 2.

### Step 2: incorporating fertility estimates in cohort component population projections

The fertility levels projected in Step 1 were incorporated into standard cohort component population projections using the Census Bureau’s Rural/Urban Projection (RUP) program. This method begins with the population of each country in 2014 (disaggregated by sex and single-year age groups) and projects it into the future based on the three components of population change – fertility, mortality, and net migration. For each country in 2014, we relied on population, fertility, and other demographic change estimates (mortality and net migration) from the Census Bureau’s International Data Base. That source was also used to project demographic change into the future (note that the IDB project changes in age-specific fertility rates in accordance with projected changes in overall fertility).

### Step 3: determining the impact of achieving the 75% benchmark by 2030

Two separate population projections were developed for each of the 13 countries: reference projections (based on United Nations’ projections of MCPR and TCPR) and benchmark projections (which assume that 75% of the United Nations’ projected demand for family planning would be met with modern methods by 2030). Differences between the projections of the reference and benchmark models in each country indicate the potential impact of achieving the family planning benchmark on the overall population as well as age structure.

## Results

Tables 2 and 3 show estimates of the projected population impact of achieving the benchmark (that 75% of demand for family planning be met through modern methods by 2030). Table 2 shows the annual growth rates of the population. In all cases, given lower fertility under the benchmark model, population growth would be lower than under the reference model, on average about 27 and 35% lower in 2030 and 2050, respectively. Table 3 shows how achieving the benchmark would affect overall population size. By 2030, expected country populations would be about 5% smaller on average. By 2050, the reduction would be larger, about 14% on average. In Nigeria, the projected % population by 2050 would be 378 million under the benchmark scenario, some 111 million (or 23%) less than the currently projected total of 489 million.

**Table 3.** Reference and 75% benchmark modern contraceptive prevalence rate estimates and projections of population size, percentages of population under age 15, and age 15–64, and youth dependency: 2014, 2030, and 2050.

	2014				2030				2050			
		Percentage				Percentage				Percentage		
Country	Total Population	Under age 15	Age 15–64	*Youth Depen- dency*	Total Population	Under age 15	Age 15–64	*Youth Depen- dency*	Total Population	Under age 15	Age 15–64	*Youth Depen- dency*
South Sudan												
Reference	11,562,602	45.8	52.1	*0.88*	19,763,787	40.8	56.4	*0.72*	32,385,679	35.2	60.3	*0.58*
75-% benchmark	11,562,602	45.8	52.1	*0.88*	18,641,344	37.3	59.8	*0.62*	26,041,695	27.1	67.2	*0.40*
DRC												
Reference	77,425,289	43.1	54.3	*0.79*	114,513,503	38.1	58.8	*0.65*	166,120,374	30.3	64.6	*0.47*
75% benchmark	77,425,289	43.1	54.3	*0.79*	105,326,203	32.8	63.9	*0.51*	132,993,986	24.1	69.6	*0.35*
Nigeria												
Reference	178,944,781	43.7	53.3	*0.82*	275,190,827	42.4	54.5	*0.78*	489,103,366	41.8	54.4	*0.77*
75% benchmark	178,944,781	43.7	53.3	*0.82*	261,939,699	39.5	57.0	*0.69*	378,309,063	31.4	63.7	*0.49*
Mali												
Reference	15,490,799	46.6	50.4	*0.92*	23,188,966	41.8	55.2	*0.76*	33,090,122	31.2	64.2	*0.49*
75% benchmark	15,490,799	46.6	50.4	*0.92*	22,041,949	38.8	58.1	*0.67*	28,082,701	26.0	68.6	*0.38*
Mozambique												
Reference	24,690,471	45.3	51.7	*0.88*	36,957,409	42.6	54.6	*0.78*	58,935,392	35.8	60.9	*0.59*
75% benchmark	24,690,471	45.3	51.7	*0.88*	35,432,190	40.1	57.0	*0.70*	49,320,111	29.1	67.0	*0.43*
Senegal												
Reference	13,493,353	42.4	54.6	*0.78*	19,472,614	36.8	59.5	*0.62*	26,981,589	28.6	65.0	*0.44*
75% benchmark	13,493,353	42.4	54.6	*0.78*	18,713,404	33.7	62.3	*0.54*	23,889,106	24.8	68.0	*0.36*
Liberia												
Reference	4,092,425	43.2	53.7	*0.80*	5,859,397	36.1	60.1	*0.60*	7,943,076	27.1	66.7	*0.41*
75% benchmark	4,092,425	43.2	53.7	*0.80*	5,578,106	32.9	63.1	*0.52*	7,086,800	24.4	68.6	*0.36*
Ghana												
Reference	25,758,052	38.6	57.3	*0.67*	35,233,423	33.5	61.1	*0.55*	47,283,540	26.6	64.7	*0.41*
75% benchmark	25,758,052	38.6	57.3	*0.67*	33,523,532	30.1	64.2	*0.47*	41,417,374	23.5	66.9	*0.35*
Afghanistan												
Reference	31,822,249	42.0	55.5	*0.76*	46,099,563	38.8	58.2	*0.67*	63,787,602	29.6	65.9	*0.45*
75% benchmark	31,822,249	42.0	55.5	*0.76*	44,265,644	36.3	60.6	*0.60*	56,341,663	25.6	69.3	*0.37*
Pakistan												
Reference	196,753,235	33.5	62.2	*0.54*	249,135,917	27.3	66.5	*0.41*	301,870,917	20.8	68.3	*0.30*
75% benchmark	196,753,235	33.5	62.2	*0.54*	241,410,568	25.0	69.0	*0.36*	278,590,913	18.4	69.8	*0.26*
Yemen												
Reference	26,074,187	41.7	55.6	*0.75*	37,189,144	33.5	62.8	*0.53*	49,177,385	24.1	68.5	*0.35*
75% benchmark	26,074,187	41.7	55.6	*0.75*	34,973,159	29.3	66.7	*0.44*	42,802,039	20.8	70.7	*0.29*
Haiti												
Reference	9,997,770	34.0	61.9	*0.55*	12,033,876	28.0	66.4	*0.42*	13,654,387	21.1	68.8	*0.31*
75% benchmark	9,997,770	34.0	62.0	*0.55*	11,373,232	23.9	70.5	*0.34*	12,207,488	18.9	70.5	*0.27*
Philippines												
Reference	107,661,003	33.7	61.8	*0.55*	138,040,714	28.2	68.6	*0.41*	169,927,915	23.0	65.2	*0.35*
75% benchmark	107,661,003	33.7	61.8	*0.55*	131,645,462	24.8	67.8	*0.37*	155,254,655	21.2	65.9	*0.32*

Given that each pair of projections begins in 2014, the expected reduction in the population under the benchmark scenario affects only those born after 2014. Figure 2 illustrates this through the population age and sex structure of the Democratic Republic of the Congo. By 2030, the projected reduction in the population is confined to those below age 15 (born 2015 to 2030), whereas by 2050, the projected reduction occurs below age 35 (born 2015 to 2050). The fertility reduction implied by achieving the 75% benchmark in 2030 (with logistic extensions) produces a population structure in 2050 less like a pyramid and more like a rectangle – the hallmark of more developed societies.

Table 3 shows the proportion of the population under age 15 implied by the reference and benchmark scenarios. The different pace and size of fertility decline under the benchmark is echoed by similar changes in the proportions of youth. In Nigeria, for example, where the projected fertility decline between 2030 and 2050 is large, the share of those below age 15 under the benchmark in 2050 is 10 percentage points lower than that of the reference model (31.4 vs 41.8%), and the youth dependency ratio – the ratio of children under age 15 to those at ages 15–64 – under the benchmark is 36% lower (.77 vs .49). On average, meeting the benchmark in these 13 countries would imply a 20% decline in youth dependency by 2050, which would portend a demographic dividend available to spur economic growth given the decline in economic ‘consumers’ (children) relative to economic ‘producers’ (working age adults) [13]. The greatest potential benefits would be to those countries with the lowest current usage of modern contraceptive methods.

## Discussion

Aforementioned results demonstrate how meeting the 75% benchmark by 2030 would affect population size, growth, and age structure in 13 developing countries. What are the broader implications of such demographic changes? In regard to lowering population size and growth, meeting the benchmark could reduce pressures on natural resources and facilitate SDGs related to environmental sustainability and food security [2]. Slower population growth makes it easier to provide sustainable infrastructure (such as clean water and sanitation), to encourage wise use of land, and to reduce pollution from industrial activities.

In addition, the lower projected proportions of youth provide an opportunity for more rapid economic development as a greater share of the population advances into economically productive ages – an opportunity known as the demographic dividend. In the East Asian context, this dividend has accounted for 10–20% of economic growth [13,21]. Lower rates of childbearing also imply greater opportunities for mothers’ education [2] and employment as well as better health for mothers and children due to broader spacing between children [3–5,22].

Some qualifications and limitations regarding these estimates deserve mention. Updated estimates and projections from the United Nations (MCPR, TCPR, and total demand for family planning) and the US Census Bureau (fertility rates and cohort component projections) would alter the estimates herein. For instance, if demand for family planning increases faster/slower than expected, meeting the 75% benchmark should have a correspondingly larger/smaller affect. Moreover, our biometric models estimated fertility by assuming a constant relative mix among modern methods and incorporate factors other than contraception (marriage and postpartum insusceptibility) based on extrapolations from the two most recent DHS studies – future studies may suggest trends other than the ones we used. All in all, however, in the absence of major changes in such parameters, our primary substantive conclusions are unlikely to change dramatically. Further details about the sources we used, the methods through which they were derived, as well as statistical and other limitations, are available [11,15,23].

## Conclusions

The indicator examined in this study – met demand for family planning – is an appealing one. Rather than specify a pre-set target of contraceptive use or an ideal family size, this indicator measures success by satisfying individual’s and couple’s own expressed desires for family planning through voluntarism and informed choice [8]. To our knowledge, this is the first study to quantify the demographic impact of achieving the family planning benchmark established by the new SDGs – to meet 75% of demand for family planning with modern contraceptive methods by 2030.

We quantified that impact by assembling demographic evidence, developing biometric models and performing population projections (reference and benchmark) for 13 countries expected to be furthest from meeting the benchmark. Although there is uncertainty as to whether the benchmark can be achieved in most developing countries [12], as well as questions about how well future demand for family planning can be predicted, there are promising examples of both developed and developing countries where rapid progress has been made, such as Belgium, Colombia, Ethiopia, France, Indonesia, Madagascar, and Rwanda [12,24]. At a minimum, the estimates herein provide an empirical framework that can facilitate discussion and prioritization of policies and programs to facilitate both a demographic dividend and progress towards family planning SDGs.

## Paper context

Many policymakers have embraced a goal that by 2030 at least 75% of the demand for family planning be met with modern contraceptive methods. This study, the first of its kind, estimates the demographic impact of meeting that goal in 13 developing countries (compared to current expectations in those countries). We summarize projected differences in future fertility rates, population size, and age structure, noting the potential demographic dividend of more rapid economic development.
